# Supplementary material for: Empirical methods for controlling false positives and estimating confidence in ChIP-Seq peaks
Source: BMC Bioinformatics. 2008 Dec 5;9:523. doi: 10.1186/1471-2105-9-523 (PMC2628906; doi:10.1186/1471-2105-9-523)
Supplement: Additional File 1 — A variety of html documents from the USeq web site detailing the available applications, their best usage, output file type descriptions, command line menus, etc. [file 1471-2105-9-523-S1.zip › USeqUserGuides/JavaDoc.zip!/JavaDoc/trans/main/class-use/Interval.html]

Uses of Class trans.main.Interval


|  |  |  |
| --- | --- | --- |
| **Overview** \| **Package** \| **Class** \| **Use** \| **Tree** \| **Deprecated** \| **Index** \| **Help** | |  |
| PREV   NEXT | **FRAMES**    **NO FRAMES**     **All Classes** |  |


---


## **Uses of Class trans.main.Interval**

| Packages that use Interval | |
| --- | --- |
| **trans.anno** | For associating intervals, binding regions, and binding peaks with GFF annotation. |
| **trans.graphics** | For graphically representing '.cel' files and intervals. |
| **trans.main** | Principle classes and scripts used in TiMAT2 analysis. |
| **trans.misc** | Rough working scripts. |

Uses of Interval in trans.anno

| Methods in trans.anno with parameters of type Interval | |
| --- | --- |
| `boolean` | `Region.intersect(Interval interval)` |
| `int` | `Region.overlap(Interval interval)`             Returns -1 for no overlap, or a positive int for the # bases of overlap. |

Constructors in trans.anno with parameters of type Interval

`BindingRegion(Interval interval,
int rank)`

Uses of Interval in trans.graphics

| Methods in trans.graphics with parameters of type Interval | |
| --- | --- |
| `void` | `IntervalDrawFrame.makePanel(Interval interval, int intervalNumber, TextFrame textFrame, boolean showPSPM)` |

Constructors in trans.graphics with parameters of type Interval

`IntervalDrawFrame(Interval interval,
int intervalNumber,
TextFrame textFrame,
boolean showPSPM)`

`IntervalDrawPanel(Interval interval,
int intervalNumber,
TextFrame textFrame,
boolean showPSPM)`

Uses of Interval in trans.main

| Methods in trans.main that return Interval | |
| --- | --- |
| `Interval` | `PosRegion.getClosestInterval()` |
| `Interval` | `PeakPicker.getInterval()` |
| `static Interval[]` | `OverlapCounter.subtractIntervals(Interval[] bigger, Interval[] smaller)`             Returns the difference between two interval arrays, be sure to get bigger and smaller correct. |

| Methods in trans.main with parameters of type Interval | |
| --- | --- |
| `static double` | `IntervalReportPrinter.aveMedianAbsDiffSubWinControl(Interval i)`             Calculate maximum median absolute diff of SubWindow control values |
| `java.lang.String` | `IntervalGraphPrinter.convertToBED(Interval[] intervals)`             Converts an array of Interval to a .bed String format. |
| `java.lang.String` | `IntervalGraphPrinter.convertToSGR(Interval[] intervals)`             Converts an array of Interval to a .sgr String format. |
| `java.lang.String` | `IntervalReportPrinter.fetchIntervalLine(Interval i, int intervalNumber)` |
| `java.lang.String` | `IntervalReportPrinter.fetchIntervalReport(Interval i, int intervalNumber)` |
| `void` | `OverlapCounter.findClosestPeaks(Interval[] one, Interval[] two, java.io.File fileOne, java.io.File fileTwo)` |
| `void` | `OverlapCounter.findClosestPeaks(Interval[] one, Interval[] two, java.io.File fileOne, java.io.File fileTwo)` |
| `static int` | `OverlapCounter.findPeak(Interval i)`             Returns base position of highest scoring peak, if no peak uses middle of sub window if no sub window uses middle of interval |
| `void` | `IntervalReportPrinter.generateIntervalStats(Interval interval)`             Calculate stats on oligos from best window. |
| `static void` | `IntervalGFFPrinter.gff(BindingPeak peak, Interval i, java.lang.String name, int peakNumber, java.lang.StringBuffer s)`             Appends a GFF3 line for a BindingPeak |
| `void` | `IntervalGFFPrinter.gff(Interval i, int rank, java.lang.String name, java.lang.StringBuffer s)`             Appends a GFF3 line for an Interval. |
| `void` | `IntervalGFFPrinter.gff(Interval i, java.lang.String name, java.lang.StringBuffer s)`             Appends a GFF3 line for a Window. |
| `static void` | `IntervalGFFPrinter.gff(Oligo o, Interval i, java.lang.String name, int oligoNumber, java.lang.StringBuffer s)`             Appends a GFF3 line for an Oligo. |
| `static void` | `IntervalGFFPrinter.gff(SubWindow sub, Interval i, java.lang.String name, java.lang.StringBuffer s)`             Appends a GFF3 line for a SubWindow. |
| `void` | `ExportIntervalData.loadBestWindowOligoIntensities(Interval interval)`             Loads the treatment and control intensity arrays for a particular interval. |
| `static double` | `IntervalReportPrinter.maxMedianAbsDiffSubWinTreatment(Interval i)`             Calculate maximum median absolute diff of SubWindow treatment values |
| `static double` | `IntervalReportPrinter.medianRatioSubWindow(Interval i)`             Calculate Median ratio of SubWindow where the treatments are averaged, and the controls are averaged |
| `boolean` | `OverlapCounter.overlap(Interval one, Interval two)`             checks to see if intervals overlap by the minimum maxGap, can set maxGap negative to require an overlap. |
| `int` | `OverlapCounter.overlapPeaks(Interval one, Interval two)`             Returns distance between top peaks. |
| `void` | `PeakPicker.pickPeaks(Interval[] intervals)` |
| `void` | `IntervalReportPrinter.printRatios(Interval interval)`             Used when making mock Intervals from which you want to calculate various oligo based measurements. |
| `static void` | `IntervalGraphPrinter.printScores(Interval[] intervals, java.io.File file, boolean printRatios)`             Prints to a File a line for every Oligo in each Interval. |
| `void` | `PosRegion.setClosestInterval(Interval closestInterval)` |
| `void` | `ScoreIntervals.setGenomicSequenceParams(Interval interval)`             Loads up the fasta file for a particular chromosome and sets some params. |
| `void` | `PeakPicker.setInterval(Interval interval)` |
| `static void` | `LoadIntervalOligoInfo.sortIntervalsByChromosome(Interval[] intervals)`             Ugly, ugly, ugly, use a Comparator! |
| `static Interval[]` | `OverlapCounter.subtractIntervals(Interval[] bigger, Interval[] smaller)`             Returns the difference between two interval arrays, be sure to get bigger and smaller correct. |
| `static Interval[]` | `OverlapCounter.subtractIntervals(Interval[] bigger, Interval[] smaller)`             Returns the difference between two interval arrays, be sure to get bigger and smaller correct. |
| `java.lang.String` | `OverlapCounter.testOverlap(Interval[] one, Interval[] two, java.io.File fileOne, java.io.File fileTwo)`             Returns the average % intersection. |
| `java.lang.String` | `OverlapCounter.testOverlap(Interval[] one, Interval[] two, java.io.File fileOne, java.io.File fileTwo)`             Returns the average % intersection. |

Uses of Interval in trans.misc

| Methods in trans.misc with parameters of type Interval | |
| --- | --- |
| `static void` | `Util.sortIntervalsBySubWindowMedianRatio(Interval[] intervals)`             Sorts an array of Interval by the median ratio of the best SubWindow. |

---


|  |  |  |
| --- | --- | --- |
| **Overview** \| **Package** \| **Class** \| **Use** \| **Tree** \| **Deprecated** \| **Index** \| **Help** | |  |
| PREV   NEXT | **FRAMES**    **NO FRAMES**     **All Classes** |  |


---
